# The Foggy Days

**Published:** 1908-10-17

**Authors:** 


### The Foggy Days.

Meteorologists tell us that we are marching to a gloomy winter, and certainly if the opening October days are to serve as an index of what is to come there is every reason to believe that this forecast will be verified. Even disregarding the first fog of the season we cannot hope that the present winter will prove less rich in murkiness than its predecessors. Under existing conditions London fogs are matters with which every city dweller will have to concern himself. In a decade perhaps we may have solved the problem of dissipating these huge clouds of dust and soot impregnated with dilute sulphuric acid by a method which does not demand a fortune for its successful execution. At present, however, known methods of dispersal are out of the bounds of practical realisation as they are either too costly or too complicated to be tried. The only hope is that, as we have stifled small-pox in these isles by vigorous prophylactic measures we may successfuly cope with soot and smoke by measures which are somewhat akin to those that we followed in dealing with the old scourge.

It is inconceivable that we should allow a state of things to continue which is costing the country yearly enormous sums of money and a sad waste of life and health. The finance of a London fog is a subject on which much might be and has been written: the pathology of it is as interesting and as sorrowful. There is no great difficulty in estimating the daily loss in pounds, shillings, and pence which the fog brings to a large commercial community through the disruption of traffic that it entails, and by the destruction of buildings, and the defilement of streets it causes. We can express it in concrete terms, in a manner that will appeal to everyone, but who shall estimate the subtler sins of a metropolitan "particular"—the incipient bronchial troubles, the lowering of vitality, and the general depressing effect on the human organism?

Year after year it is pointed out that fog, like small-pox and zymotic disease, is preventable if not curable, and year after year we are treated to a new hypothesis with regard to its dispersal; yet no one hears anything further about prophylactic measures. There exist excellent regulations which are more drastic, we believe, than those which were made against variola. The smoke spreading manufacturer whose chimney defiles what Gordon called in capitals, God's Glorious Oxygen, cannot shelter himself behind a "conscientious objector" clause. He cannot point out that the regulations which forbid him doing what he does daily are founded on "mere hypotheses" as the vaccinator can; he cannot invoke a smattering of science or pervert a series of statistics to confound us, and he does not need the "argument of Leicester" to convince him of his errors. The law is definite for him, and yet as far as London and most of our large cities are concerned it is as much a dead letter as our vaccination legislation promises to be when Mr. John Burns has done with it. That this should be so is a matter of grave regret. The medical profession, that sees more of the evil effects of fog upon the community than most people do, is as much concerned in dealing with its suppression as it is concerned in the enforcement of the vaccination laws. To it belongs the duty of rousing public opinion on the subject, of creating a movement that shall result in the rigid enforcement of existing laws against the pollution of the atmosphere. The societies for the abatement of the smoke nuisance may help in the matter by acquainting the public with the hygienic dangers and terrors of a foggy day. The statistics which are needed for drawing up a full indictment, from the medico-social point of view, against the fog, are not yet available. They demand some care and research, and a definite comparison of mortality figures, and, what is more important and much more difficult, of disease figures between the average winter's day and the foggy day. There can be no doubt that such a compilation will prove profoundly interesting, and its publication will at least serve a useful purpose in drawing attention to our annual horror which is to a large extent preventable.

